# Normalization of RNA-Seq data using adaptive trimmed mean with multi-reference

**DOI:** 10.1093/bib/bbae241

**Published:** 2024-05-20

**Authors:** Vikas Singh, Nikhil Kirtipal, Byeongsop Song, Sunjae Lee

**Affiliations:** School of Life Sciences, Gwangju Institute of Science and Technology, 123 Cheomdan-gwagiro, 61005, Gwangju, South Korea; School of Life Sciences, Gwangju Institute of Science and Technology, 123 Cheomdan-gwagiro, 61005, Gwangju, South Korea; School of Life Sciences, Gwangju Institute of Science and Technology, 123 Cheomdan-gwagiro, 61005, Gwangju, South Korea; School of Life Sciences, Gwangju Institute of Science and Technology, 123 Cheomdan-gwagiro, 61005, Gwangju, South Korea

**Keywords:** RNA-seq, α trimmed mean, normalization, differential expression, AUC, jaeckel’s estimator

## Abstract

The normalization of RNA sequencing data is a primary step for downstream analysis. The most popular method used for the normalization is the trimmed mean of M values (TMM) and DESeq. The TMM tries to trim away extreme log fold changes of the data to normalize the raw read counts based on the remaining non-deferentially expressed genes. However, the major problem with the TMM is that the values of trimming factor M are heuristic. This paper tries to estimate the adaptive value of M in TMM based on Jaeckel’s Estimator, and each sample acts as a reference to find the scale factor of each sample. The presented approach is validated on SEQC, MAQC2, MAQC3, PICKRELL and two simulated datasets with two-group and three-group conditions by varying the percentage of differential expression and the number of replicates. The performance of the present approach is compared with various state-of-the-art methods, and it is better in terms of area under the receiver operating characteristic curve and differential expression.

## Introduction

High-throughput RNA-seq is one of the most effective tools for investigating various biological and medical applications. Highly complex and massive data sets generated by sequencers initiate a need to develop statistical and computational data analysis methods [1, 2]. In RNA-Seq, the RNA is fragmented and reverse-transcribed to complementary DNA or vice versa. These fragments are sequenced, and produce reads aligned to a pre-sequenced reference genome or transcriptome or, in some cases, assembled without the reference. These reads are mapped to a gene and used to quantify its expression [3]. The raw read counts have a different source of systematic variation, which includes differences between samples, such as library size [4] or differences within samples, gene length [5] and guanine–cytosine (GC) content [6]. These variations affect the differential expression (DE) analysis of the RNA-seq data. It can be overcome by a suitable normalization method similar to microarray-based gene expression data analysis [7, 8]. The authors [9, 10] have described how normalization affects the differential gene expression analyses in microarray data. Arguably, the choice of the normalization method can significantly affect the downstream analysis results more than the method used for completing DE [9].

Normalization is classified into two categories: within-sample and between-sample. Within-sample normalization helps to correct the expression level of each gene associated with the expression level of other genes in the same sample. The most broadly studied methods for within-sample and between-sample normalization are Reads Per Kilobase Million (RPKM) [4] and Fragments Per Kilobase Million (FPKM) [11]. However, the different gene lengths can lead to a bias in per-gene variance for DE analysis of low-abundance genes [4]. In the literature, within-sample normalization approaches for RNA-seq data have corrected the biases arising from library size, gene length, and GC content. These normalization methods are Total Counts (TC) [12], Upper Quartile (UQ) [10, 13], smooth quantile normalization [14], per-sample Median (Med) [10], DESeq normalization [15], trimmed mean of M values (TMM) [16], Tag Count Comparison [17] and sequencing data based on a Poisson log-linear model [18]. To correct the library size, TC, UQ, Med, DESeq, and TMM are used, and they are based on a common normalizing factor per sample to normalize the genes. Selecting the optimal method from the perspective of sensitivity and specificity will be challenging due to biological variation, read depth, and the number of biological replicates in the RNA-seq data [19]. Based on the DE analysis, the previous studies suggested that the DESeq and TMM perform better than the other methods [20–22]. The normalization of RNA-seq data using factor analysis of control genes or samples and transformation techniques for constructing gene coexpression networks from RNA-seq data are discussed in [23, 24]. A comprehensive evaluation of normalization methods for Illumina high-throughput RNA sequencing data is discussed in [25, 26]. In recent studies, the authors have utilized the TMM to normalize the data to find the differential abundance and predict preeclampsia in pregnancy [27, 28].

In bulk RNA-seq, the data may vary based on various uncontrollable experimental conditions. RNA-seq raw data must be normalized to scale the read counts. The most widely used and well-accepted methods for bulk RNA-seq data analysis are the TMM and DESeq ([29]). In the TMM, trimmed factor values are heuristic or user-defined. In this paper, we have presented an adaptive approach that selects the trimming factor from data automatically. The value of the trimming factor is estimated from the data using Jaeckel’s estimator, which helps to find a more robust factor by minimizing the asymptotic variance estimate of the alpha-trimmed mean. Additionally, in the TMM, only one sample from the data acts as a reference signal to find the value of the scale factor, which may be biased. To overcome this effect, we have used all the samples as reference signals to find the common scale factor of the samples.

The rest of the paper is organized into four sections. Section 2 discusses the present method. Experimentation and performance evaluation are described in Section 3. Section 4 briefly concludes the paper.

The key contributions of the paper are briefly described as follows:

- The value of the trimming factor is estimated from the data using Jaeckel’s estimator, which offers a robust trimming factor to trim away the extreme log fold changes. The trimming factor is obtained by minimizing the asymptotic variance estimate of the alpha-trimmed mean estimator.
- TMM uses only one sample from the data as a reference signal to find the scale factor, which may be biased. To overcome this effect, we have used all the samples as reference signals to determine the scale factor. We get the scale factor matrix of order $n*n$ and apply the geometric mean corresponding to the column to find the common scale factor.

## Proposed approach: adaptive trimmed mean of M

As described [16], the trimmed mean is the average after trimming the upper and lower values (x%) of extreme log fold changes. The TMM method is dual-trimmed by log fold changes ($M_{g}$) ($k^{th}$ sample relative to $r^{th}$ sample for gene g) and by absolute expression intensity ($A_{g}$ ). The default trimming factor for $M_{g}$ is 30 %, and for $A_{g},$ it is 5% [16]. The gene-wise log fold changes and absolute expression intensities for sequencing data are defined as follows: 


(1) \begin{align*} M_{g} =&\ \frac{log_{2}\Big(\frac{Y_{gk}}{N_{k}}\Big)}{log_{2}\Big(\frac{Y_{gr}}{N_{r}}\Big)} \nonumber\\ A_{g} =&\ \frac{1}{2}log_{2}\left(\frac{Y_{gk}}{N_{k}}\times\frac{Y_{gr}}{N_{r}}\right),\ \text{for}\ Y_{gk}, Y_{gr} \neq 0 ,\end{align*}

where, $Y_{gk}$ and $Y_{gr}$ are the observed read count of gene $g$ with respect to sample $k$ and reference sample $r$, and $N_{k}$ and $N_{r}$ are the total number of raw read counts for sample $k$ and $r$, respectively.

To robustly summarize the observed $M_{g}$ values, the authors have trimmed both the $M_{g}$ and $A_{g}$ values before taking the weighted average. The weights are utilized to account for the fact that log fold changes from genes with large read counts have small variances on the logarithm scale [16]. As explained, the normalization factor for sample $k$ using reference sample $r$ is determined as 


(2) \begin{align*}& \log_{2}(TMM_{k}^{r})= \frac{\sum_{{g}\in G^{*}} w_{gk}^{r}M_{gk}^{r}}{\sum_{{g}\in G^{*}} w_{gk}^{r}} ,\end{align*}

where 


(3) \begin{align*} M_{gk}^{r} &= \frac{log_{2}\Big(\frac{Y_{gk}}{N_{k}}\Big)}{log_{2}\Big(\frac{Y_{gr}}{N_{r}}\Big)} \nonumber\\ w_{gk}^{r} &= \frac{N_{k}- Y_{gk}}{\frac{N_{k}}{Y_{gk}}} + \frac{N_{r}- Y_{gr}}{\frac{N_{r}}{Y_{gr}}},\ \text{for}\ \ Y_{gk}, Y_{g}> 0 ,\end{align*}

where $w_{gk}^{r}$ is the weight as the inverse of the asymptotic variance.

The TMM performs well, but the key issue is selecting the optimal trimming factor value. To get the optimal values of trimming factor we have used the asymptotic properties of Trimmed Means.

Let $X_{1}, X_{2},...,X_{n}$ be a sample of independent, identically distributed (iid) with cumulative distribution $E(\alpha )$. The $X$ may be the $M_{g}$ or $A_{g}$ values. The alpha-trimmed mean is given as 


(4) \begin{align*}& \mu_{n}(\alpha) = \frac{1}{n-2[\alpha n]}\sum_{i=[\alpha n] +1}^{i = n- [\alpha n]} X_{i}\end{align*}

With the assumption that $E^{-1}(\alpha )$ and $E^{-1}(1 -\alpha )$ are unique, it is shown [30, 31] that Equation (4) is an asymptotically normal estimator with respect to sample asymptotic variance estimate ($V(\alpha )$), i.e. 


(5) \begin{align*}& n^{\frac{1}{2}}\{\mu_{n}(\alpha) - \mu(\alpha) \} \xrightarrow{D} N(0, V(\alpha)) ,\end{align*}

where 


(6) \begin{align*} \mu(\alpha) =&\ \frac{1}{1-2\alpha} \int_{E^{-1}(\alpha)}^{E^{-1}(1 -\alpha)} xdE(x) \nonumber\\ V(\alpha) =&\ \frac{1}{(1- 2\alpha)^{2}} \times \biggl\{ \int_{E^{-1}(\alpha)}^{E^{-1}(1 -\alpha)} (x - \mu(\alpha)^{2}dE(x) \nonumber\\& +\alpha (E^{-1}(\alpha) - \mu(\alpha))^{2} +\alpha (E^{-1}(1- \alpha) - \mu(\alpha))^{2} \biggl \} \end{align*}

The asymptotic alpha-trimmed mean estimator ($\mu (\alpha $)) in Equation (4) is optimized by selecting an $\alpha _{opt}$ such that 


(7) \begin{align*}& \alpha_{opt} = \arg\min V(\alpha)\end{align*}

The continuous form of asymptotic variance can be written in discrete form using the Jaeckel’s Estimator as follows: 


(8) \begin{align*}& \begin{aligned} V_{n}(\alpha) = \frac{1}{(1-2\alpha)^{2}}\times \left\{ \frac{1}{n} \sum_{i=[\alpha n] +1}^{i = n- [\alpha n]} (x_{i} - \mu_{n}(\alpha))^{2}\right. \\ +\ \alpha(x_{i=[\alpha n] +1}(i) - \mu_{n}(\alpha))^{2} \\ +\ \alpha(x_{n- [\alpha n]}(i) - \mu_{n}(\alpha))^{2} \Biggl \} \end{aligned}\end{align*}

The optimal values of trimming factor ($\alpha $) is obtained as 


(9) \begin{align*}& \alpha^{opt} = \arg \min \{V_{n}(\alpha): 0 \leq \alpha < \delta \}\end{align*}

**Figure 1:**
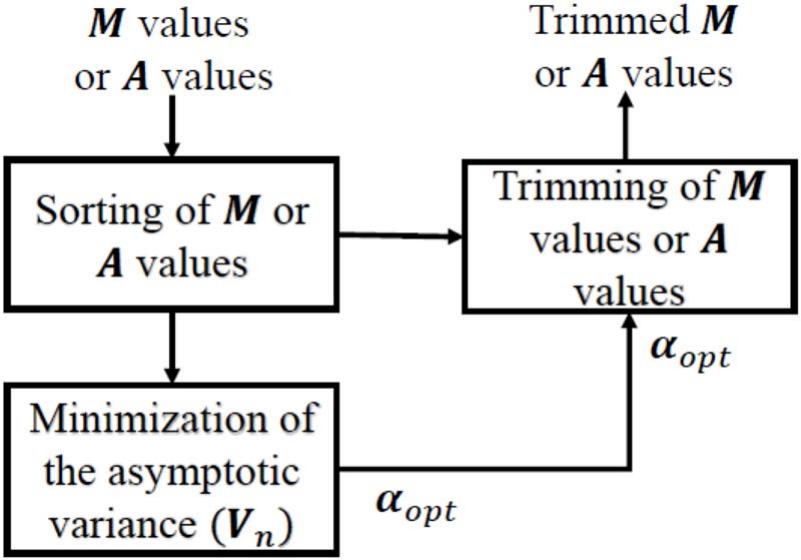
Pictorial representation of learning $\alpha _{opt}$ using Jaeckel’s estimator for trimming of $M$ or $A$.

The default value of $\delta $ is $0.5$ and the proposed approach is graphically described in Fig. 1, and explained in algorithm. 


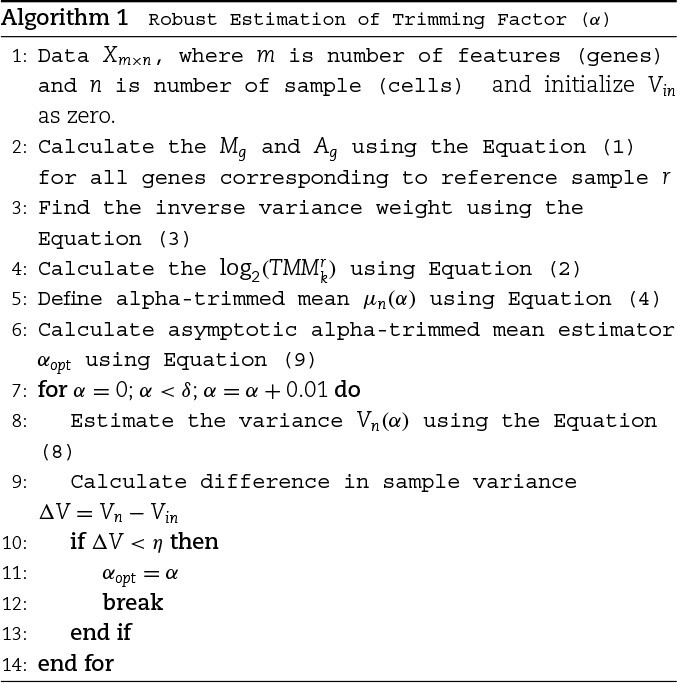


## Results and discussion

This section described the detailed description of datasets and performance evaluation of the normalization methods.

### Real datasets

#### Sequencing quality control dataset

The performance of normalization methods is examined on the RNA-Seq dataset of the sequencing quality control (SEQC) project [32]. This project described RNA-Seq technology across distinct platforms and alignment methods and collected the dataset of four samples, i.e., A, B, C, and D, with the number of replicates per sample [32]. The SEQC dataset also has TaqMan quantitative real-time polymerase chain reaction (qRT-PCR) measurements on 1000 genes. The PCR data are commonly used to identify true differential gene expression and determine false negatives and positives in RNA-Seq data. The complete SEQC qRT-PCR data have 1044 genes. Here, we also perform a similar study as presented by authors [3], in which the PCR data are matched with SEQC RNA-Seq data. They have selected common genes with enough information and eliminated duplicate genes. The unique genes obtained from both RNA-Seq and PCR measurements are 733 genes.

#### Microarray quality control project dataset

In the second study, we analyzed the performance of each method on the MAQC2 and MAQC3 datasets of the microarray quality control project (MAQC) Project [33]. The MAQC2 has two RNA-Seq datasets from the MAQC project with two distinct biological samples, i.e., human brain reference RNA (hbr) and universal human reference RNA (uhr). The MAQC2 is accessed from the NCBI sequence read archive with reference ID SRX016359 (hbr) and SRX016367 (uhr), and it consists of a read length of 36bp [10] and second dataset (GEO series GSE24284) consists of the 50bp hbr (sample ID: GSM597210) and uhr (sample ID: GSM597211) RNA samples ([34]). MAQC3 is accessed from GEO (GSE49712), and it has five technical replicates in two biological conditions (uhr and hbr) [35].

#### Pickrell dataset

Pickrell’s real data are accessed from the recount2 database with the sample ID ‘SRP001540’. It has an order of count matrix $58\,037 \times 160$ of human data generated from two sequencing centers, i.e., Yale and Argonne [36, 37]. The dataset obtained from both centers shows similar results [38], so we perform normalization on the Yale dataset with 79 samples. The column matrix of the dataset is reduced by summing the samples with technical replicates, which results in 69 samples and genes with a zero count value in all samples being removed, and the analyzed genes are 51, 910. The analyzed Pickrell dataset consists of a count matrix $51\,910 \times 69$ that compares the expression levels of lymphoblastoid cells between 29 males and 40 females.

### Simulated datasets

The effectiveness of the normalization methods is also validated on the simulated data by varying the proportion of DEGs and DEGs up-regulated in the individual conditions. The simulated data of two and three-group conditions are generated with the help of the simulateReadCounts function of the TCC package in R [39].

#### Two-group comparison

The two-group simulated data consist of g = 10 000 genes. The number of simulated biological replicates of individual groups is $r_{1} = r_{2} =3$, the proportion of DEGs (PDEG = 0.25), DEGs up-regulated in the individual conditions are $P_{1} = 0.9$ (or $P_{2} = 0.1$), and degree of DEGs is fixed at 4-fold (FC = 4).

#### Three-group comparison

As described in [40], we also validated on three-group simulated data where the number of simulated biological replicates is $r_{1} = r_{2} = r_{3} = 3$. The simulated conditions are g = 10 000, the proportion of DEGs (PDEG = 0.25), and the degree of DEGs is fixed 4-fold (FC = 4). Here, we generated two conditions for each group with the proportion of up-regulated DEGs (1/3, 1/3, 1/3) and (0.4, 0.2, 0.4) [41].

### Performance evaluation

#### Performance analysis on real datasets

In this study, the proposed approach is compared with five widely used normalization methods, i.e., DESeq [15], PoissonSeq [18], DEGES [17], SQ [14], and TMM [16] on real datasets. Here, we briefly describe the libraries used for the normalization and statistical tests. edgeR [13] performs TMM normalization based on the Bayes estimation and the exact test with a negative binomial distribution for DE genes analysis. DESeq2 uses median-based normalization to account for the presence of different library sizes. DESeq2 estimates the gene-wise dispersions and shrinks these estimates to generate more accurate dispersion estimates to model the counts. Finally, it fits the negative binomial distribution model and performs hypothesis tests using the Wald or Likelihood Ratio tests. TCC [39] is a multi-step normalization method (called DEGES) that uses inbuilt functions of other libraries, i.e., DESeq2, edgeR, and so on. PoissonSeq, another software used in our analysis, is based on an iterative process. It estimates a group of non-DE genes, and the scaling factor of each sample is determined using the respective group. In this study, we have performed a Wald test to find the differentially expressed genes for DESeq2, DEGES, and PoissonSeq.

**Figure 2:**
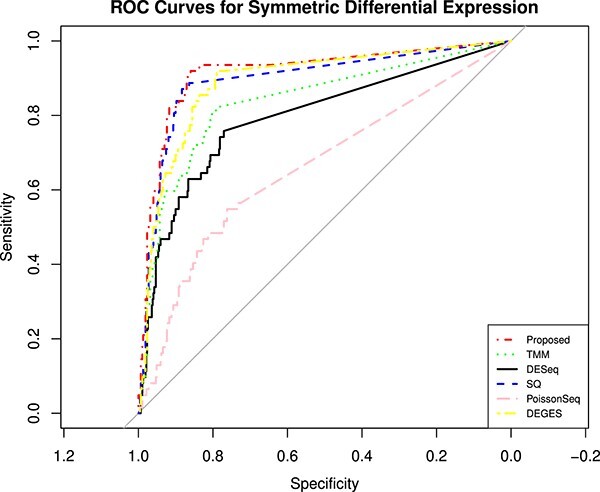
ROC curves for symmetrical DEGs on SEQC data using each normalization method. The figure shows the ROC performance on RNA-Seq data with 733 PCR-validated genes.

**Figure 3:**
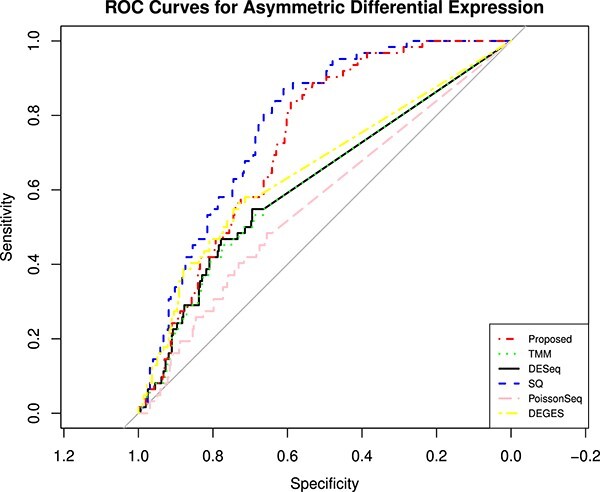
ROC curves for asymmetrical DEGs on SEQC data using each normalization. The figure shows the ROC performance on RNA-Seq data with 619 PCR-validated genes.

Genes with small read counts across the libraries have very little information for DE. These genes are filtered automatically using the edgeR function filterByExpr. After removing the low-expressed genes, the data are normalized using different normalization methods. For the SEQC data, we have performed two tests, first for symmetric expression and the other for asymmetric expression, and the performance is compared in terms of area under the receiver operating characteristic curve (AUC) values. As shown (Fig. 2) for symmetrical DEG analysis, if the data using a complete set of 733 genes are normalized using the proposed approach, it performs better than other methods. However, in the case of asymmetric expression, the proposed method also performs a little better except for smooth quantile normalization (Fig. 3). The smooth quantile normalization normalized performs slightly better for asymmetrical DE since the data are normalized using linear assumption. At each quantile, a linear model is fitted to find the covariate, and the normalized data are obtained by taking the weighted average of each quantile. To analyze the performance of normalization methods under asymmetric expression, we have taken a subset of 619 PCR-validated genes with $75\%$ of DE genes up-regulated in sample A, and the rest $25\%$ of DE genes are up-regulated in sample B. We have also shown the false discovery rate (FDR) values (Table 1) in the case of two and five technical replicates. The AUC is used to compare the results of all methods based on the log-fold-change $<= 0.5$.

**Table 1:** FDR with standard deviation (SD) for RNA-seq dataset of SEQC project with two and five replicates per sample

replicates	DESeq [15]	DEGES [17]	PoissonSeq [18]	TMM [16]	SQ [14]	Proposed
	FDR(SD)	FDR(SD)	FDR(SD)	FDR(SD)	FDR(SD)	FDR(SD)
2	$0.054(0.004)$	$0.052(0.005)$	$0.073(0.005)$	$ 0.051(0.004)$	0.059(0.005)	$0.051(0.004)$
5	$ 0.072(0.003)$	$ 0.082(0.002)$	$0.06(0.003)$	$0.071(0.003)$	0.072 (0.003)	$0.069(0.002)$

**Figure 4:**
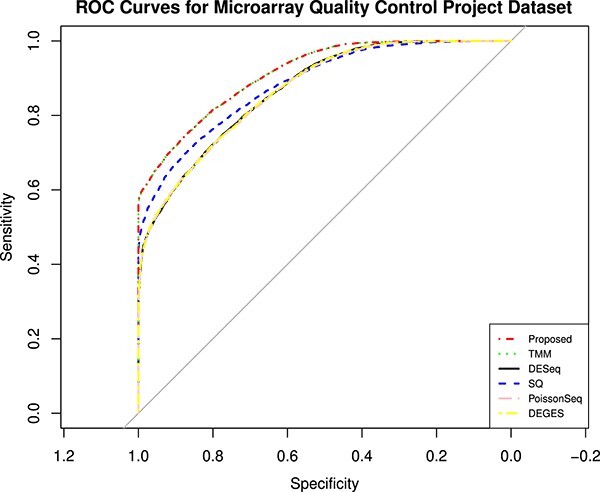
The ROC curves showing the performance of five normalization methods on MAQC2 dataset with two technical replicates in two-group condition.

**Figure 5:**
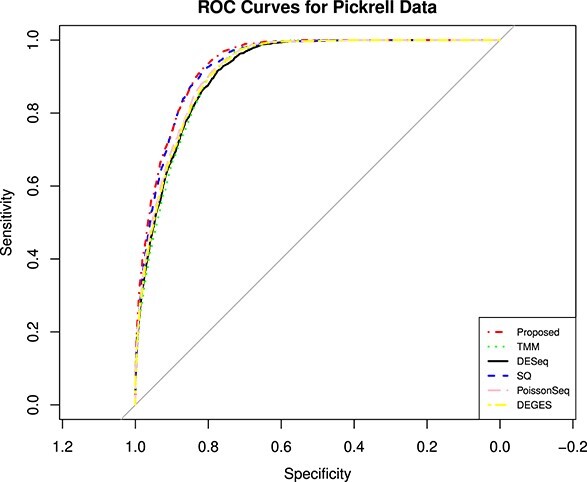
The ROC curves describing the performance of five normalization methods on two-group condition of Pickrell data.

**Figure 6:**
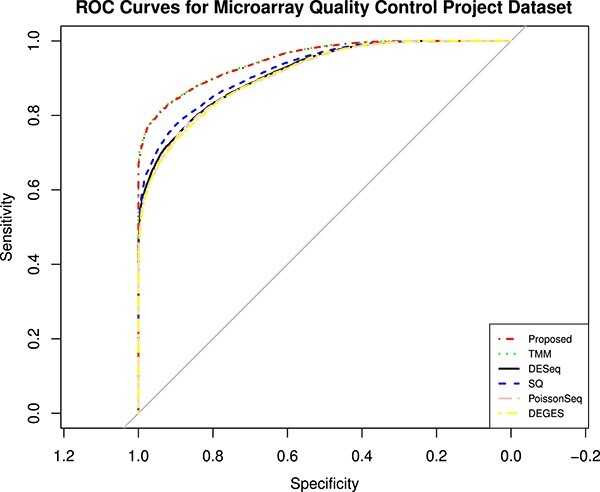
The ROC curves showing the performance of five normalization methods on MAQC3 dataset with five technical replicates in two-group condition.

The MAQC data have two datasets, i.e., MAQC2, which has two replicates, and MAQC3, which has five replicates. The genes with low counts are filtered out similarly to the first datasets using the edgeR function filterByExpr. The filtered data are normalized using different normalization methods and DEs, and statistical tests are performed. Figures 4 and 6 show the AUC curve of different approaches. The average AUC values are also presented in Table 2, which shows that the proposed method is better than the rest. We also examined the performance of the presented method on the two-group Pickrell dataset. After removing the genes with low counts, the data are normalized using all the methods. The DE results on the normalized data are 161 DE genes in the proposed approach, 162 in TMM, 53 in DESeq2, 48 in DEGES, and 45 in PoissonSeq. As shown (Fig. 5) on the Pickrell data, the AUC plot indicates that the present approach can find genes classified between the two classes better than the other methods. The AUC values are also given (Table 2) for better comparison. We have also plotted the Venn diagram plot of the differentially expressed genes on the Pickrell dataset, as shown in Fig. 8. The MA plot on the Pickrell dataset shows how many DE genes are identified by the proposed and state-of-the-art methods using the test for DE for RNA-seq data (XBSeq) [42]. The MA plot (Fig. 9) also shows the common DE genes between the present and other state-of-the-art methods.

**Figure 7:**
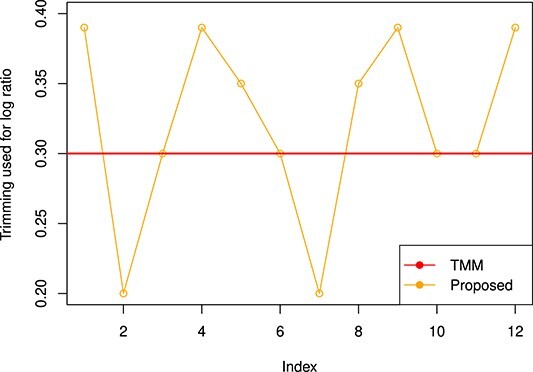
Trimming factor values are plotted for the MAQC2 dataset with proposed approach (orange line) with TMM (red line).

**Figure 8:**
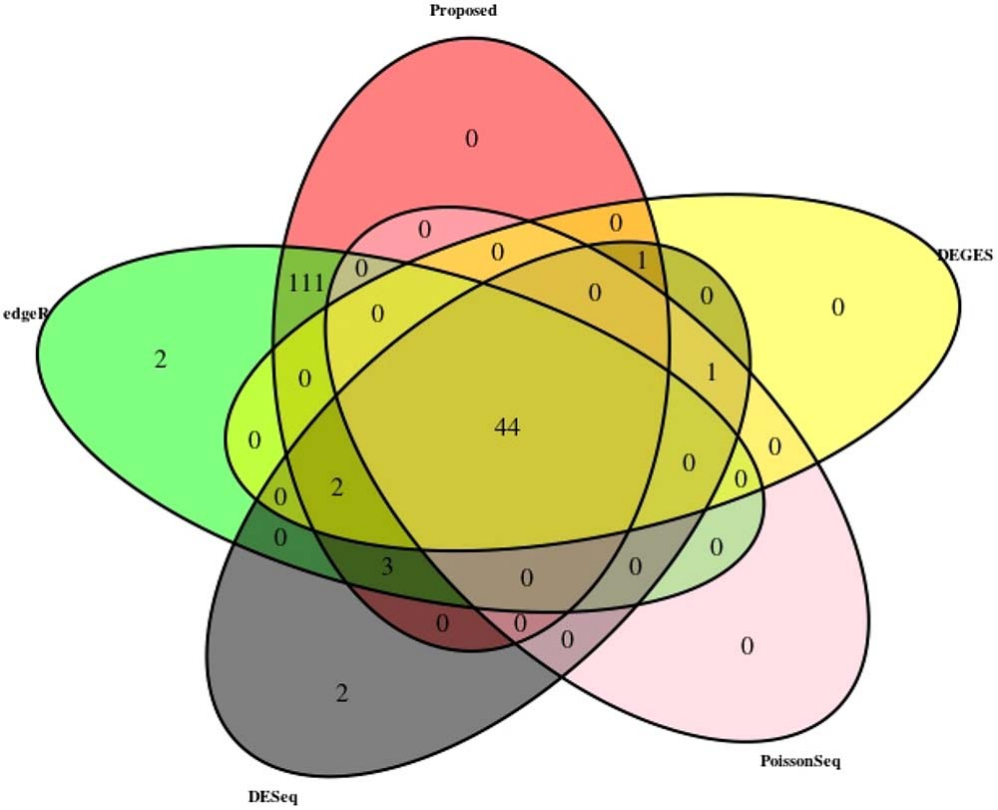
Venn Diagram on Pickrell dataset with pval$<0.01$ and abs(log-fold-change)$>1$.

**Table 2:** Comparison of AUC values on real datasets with state-of-the-arts

Datasets		DESeq [15]	DEGES [17]	PoissonSeq [18]	TMM [16]	SQ [14]	Proposed
Real Data	SEQC (SDE)	79.38	89.08	66.02	83.69	89.46	**91.52**
	SEQC (ADE)	62.09	64.54	56.67	62.01	77.93	**73.34**
	MAQC2	86.81	86.79	86.68	91.30	88.29	**91.31**
	MAQC3	91.47	91.10	91.16	94.07	92.35	**94.98**
	Pickrell	91.66	92.01	92.34	91.56	92.90	**93.66**

SDE: Symmetric differential expression, ADE: Asymmetric differential expression

#### Performance analysis on simulated datasets

The performance of the proposed approach has been experimented on the simulated datasets with two- and three-group conditions in terms of AUC values by varying the percentage of DEGs and up-regulated genes using the TCC function simulatedReadCounts. The AUC value provides data comparisons without a trade−off in specificity and sensitivity. The ROC curve depicts the true positive rate (i.e., sensitivity) versus the false positive rate (1− specificity) obtained for each threshold condition. In the two-group comparison, we have varied the DEGs from $5\%$, $15\%$ and $35\%$, respectively, and up-regulated genes are varied from $5\%$, $15\%$, $25\%$ and $35\%$ as shown (Figs. 10, 11, and 12). However, in the case of three-group generated data, we have varied the DEGs from $5\%$, $15\%$, $25\%$, $35\%$, $55\%$ and $65\%$, respectively, with proportion of up-regulated genes varied from $33.33 \%$ in each group as shown (Fig. 13) and $40 \%$, $20 \%$ and $40 \%$ as shown (Fig. 14). The present approach performs better due to the variable trimming percentage of log-fold change.

**Figure 9:**
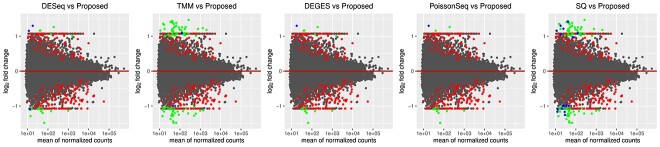
The red dots indicate DE genes identified only by the proposed method. The green dots are the shared results of proposed method and other state-of-the-art methods. The blue dots are DE genes identified only by state-of-the-art on the Pickrell dataset.

**Figure 10:**
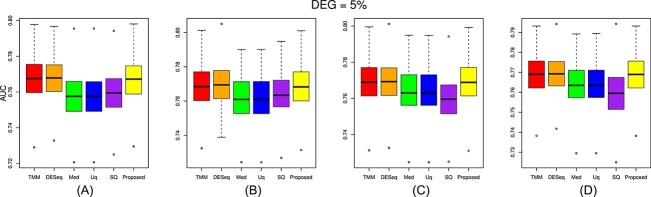
Two-group simulated data have 5% deferentially expressed genes and each group has three replicates with up-regulated deferentially expressed genes varied from (A) 5%, (B) 15%, (C) 25%, (D) 35% in each group.

**Figure 11:**
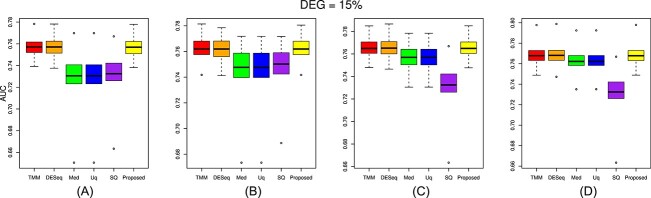
Two-group simulated data have 15% deferentially expressed genes and each group has three replicates with up-regulated deferentially expressed genes varied from (A) 5%, (B) 15%, (C) 25%, (D) 35% in each group.

**Figure 12:**
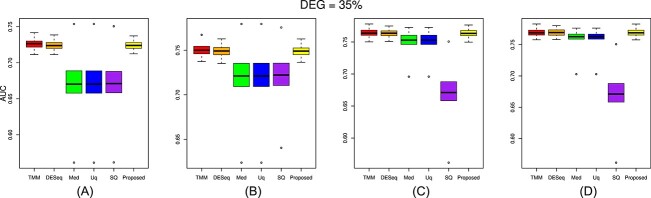
Two-group simulated data have 35% deferentially expressed genes and each group has three replicates with up-regulated deferentially expressed genes varied from (A) 5%, (B) 15%, (C) 25%, (D) 35% in each group.

**Figure 13:**
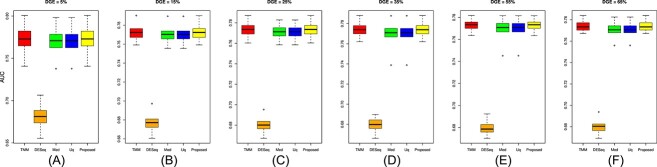
Three-group simulated data with deferentially expressed genes are (A) 5%, (B) 15%, (C) 25%, (D) 35%, (E) 55%, (F) 65% and each group has three replicates with up-regulated deferentially expressed genes 33.33% in each group, respectively.

#### Trimming factor

The trimming factor is an essential parameter for trimming the data while preserving the desired information for the DEG analysis. It is estimated using Jaeckel’s Estimator and plotted (Fig. 7). As shown in the figure; we are getting different trimming values (change from 0.20 to 0.39 in case of MAQC2 dataset) for log fold change compared with TMM (by default, 0.30), which helps to find a better scaling factor while normalizing the samples.

**Figure 14:**
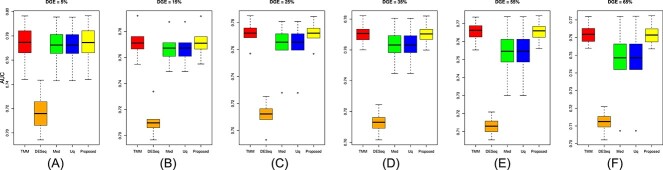
Three-group simulated data with deferentially expressed genes are (A) 5%, (B) 15%, (C) 25%, (D) 35%, (E) 55%, (F) 65% and each group has three replicates with up-regulated deferentially expressed genes 40% in group 1, 20 % in group 2 and 40% in group 3, respectively.

#### Computational performance

We compared the computational performance of the present approach with five normalization methods on real datasets, as shown (Table 3). The execution time of the present approach is comparable for datasets with a small number of samples. In the case of datasets with a large sample size, all samples are used as references to find the better scale factor. We get the scale factor matrix across all samples with an order of $n*n$ and apply the geometric mean corresponding to the column to find the common scale factor, which increases the execution time with improved performance.

**Table 3:** Comparison of runtime (in seconds) on real datasets with state-of-the-arts

Datasets	DESeq [15]	DEGES [17]	PoissonSeq [18]	TMM [16]	SQ [14]	Proposed
SEQC (SDE)	1.99	4.61	2.92	1.71	1.80	53.92
SEQC (ADE)	1.81	4.39	2.89	1.57	1.85	51.14
MAQC2	3.73	11.76	4.60	2.70	2.93	3.11
MAQC3	7.24	18.71	7.11	2.70	4.60	7.03
Pickrell	21.41	100.21	23.29	17.23	24.86	78.80

## Conclusion

This paper presents an adaptive approach for bulk RNA-Seq data normalization. It automatically selects the trimming value of $M$ corresponding to the log fold change and absolute mean expression of RNA-Seq data to calculate the size factor for normalizing the data. It validated the real and simulated datasets to identify their effectiveness compared with state-of-the-art methods. Table 2 shows the effectiveness of the present approach on the real datasets. In the simulated datasets, as we varied the percentage of DE genes and up-regulated DE genes in the two- and three-group conditions, the proposed method performed better, as described in the performance evaluation subsection and in Figs. 10, 11, 12, 13 and 14, respectively. The effectiveness in terms of performance shows that the present approach scales the datasets better than the rest of the methods in most cases since it trims each sample with different trimming values. The proposed approach will be extended to single-cell dataset normalization in the future.

**Key Points**

- This study presents a novel normalization approach of bulk-RNA-Seq data with improved performance.
- The $M$ and $A$ values are automatically trimmed according to datasets.
- The present approach can find better differential expressed genes compared with state-of-the-art methods.
- The present approach utilized all the samples as references to get the scale factor of the sample, which removes the biases due to individual samples as references in the TMM.

## Author Biographies

**Vikas Singh** received his PhD in the Department of Electrical Engineering, Indian Institute of Technology Kanpur, India. He is working as a postdoctoral research associate in the School of Life Science at Gwangju Institute of Science and Technology, South Korea. His research interests are single and bulk cell data Analysis, spatial transcriptomics data analysis, genome-scale modeling, bioinformatics, image processing, machine learning and deep learning, and fuzzy logic systems.

**Nikhil Kirtipal** has a PhD in biotechnology and expertise in molecular genetics in population studies and NGS data analysis. He intends to expand my scientific understanding and approaches.

**Byeongsop Song** is working toward the doctoral program at Pusan National University, South Korea. Currently, he is working as a researcher in the Life Mining Lab at Gwangju Institute of Science and Technology, South Korea, and analyzing Korean IBD samples. His specific interest area is Robust Statistics and Bayesian Modeling.

**Sunjae Lee** is a lead scientist in datadriven life sciences, such as systems biology of the human microbiome and the metabolism. He has received a PhD in bioinformatics/data mining at KAIST and has had professional experiences in world-leading systems biology groups of humans and microbiomes in Sweden and the UK. He is leading the group of life data mining at Gwangju Institute of Science and Technology, South Korea, and investigating many types of chronic diseases where microbiome and metabolism matter.
